# Missing Parts in *The Shoemaker’s Holiday*

**DOI:** 10.1086/694329

**Published:** 2017

**Authors:** Alanna Skuse

**Affiliations:** University of Reading

These silken fellows are painted images—outsides, outsides, Rose, their inner linings are torn.

(11:40–41)^1^

In 1599, Philip Henslowe recorded in his diary that he had paid “thomas dicker” the sum of three pounds for a play about “the gentle craft”; that is, about shoemaking.^2^ This play, which became *The Shoemaker*’*s Holiday*, reworked Thomas Deloney’s 1598 *The Gentle Craft*, building on its story of Simon Eyre, the London shoemaker who becomes Lord Mayor, and adding the new subplot of injured shoemaker Ralph/Rafe and his lost wife Jane.^3^ The play would have been performed sometime in 1599, and seemingly played well, since it was acted by the Admiral’s Men for Queen Elizabeth on New Year’s Day, 1600.^4^

Despite sounding, and feeling, very much of its own time, *The Shoemaker*’*s Holiday* has remained among the most popular of the era’s citizen comedies, with several twentieth- and twenty-first-century productions, and a substantial body of criticism devoted to the play’s structure,^5^ characterization of the “gentle craft,”^6^ and relationship to Shakespeare’s *Henry V*.^7^ In recent years, scholars have emphasized the play’s obsession with commercialism, material goods, and craftsmanship.^8^ Meanwhile, the matter of journeyman Ralph’s “lame” form has attracted relatively little critical attention.^9^ However, this essay will demonstrate that Ralph’s missing or injured leg and the plethora of material goods that populate the rest of the play are not divorced concerns, but closely wedded.

I argue that in *The Shoemaker*’*s Holiday*, Dekker shows himself to be interested in prostheses in the broad sense of that word. He shows how various characters rely on clothing and material goods not only to express, but actually to construct their identity, such that the boundary between person and thing becomes indistinct. However, the play also shows that this kind of identity is not the only or the best model. Ralph, whose disability renders him in need of prosthesis in the traditional sense, is unable to participate in this material putting-on of identity. He cannot shed his lameness, of which the economic and social significance is so great that his friends need not, and dare not, acknowledge it. Unable to assume a different identity based on the sumptuary “transformation” of his body, he must embrace an alternative ontology. As Dekker shows, there is a less showy but nonetheless valuable place in the world to be established via the bonds of matrimony and fraternity, and the enduring value of artisanal skill. This identity is no more an “essential self” than that of the man “made” by clothes, but it is reliable, self-directed, and inalienable. Finally, I will show that even as Dekker makes his argument for reconstructing identity in terms of interpersonal relationships, he complicates and interrogates that model. Ralph’s reintegration into the shoemakers’ society is promising, but not complete. Moreover, the fact that Ralph’s disability is “put on” by an able-bodied actor in front of a (potentially impaired) audience returns us once more to the problematics of physicality, identity, properties, and prosthetics.

## Making Identity from Properties and Prosthetics

*The Shoemaker*’*s Holiday* is, in its simplest form, a dream of upward mobility; of nice clothes, parties, and good things to eat and drink. The play is filled with characters seeking to move between different social and economic modes: Lacy, who seeks to circumvent the aristocratic principles that prevent him from wedding Rose; Firk and Hodges, riding on the coattails of their rapidly rising master; and Simon and Margery Eyre, whose canny use of labor allows them to move into the mercantile world, trading in shipments and holding administrative office.^10^ Ralph’s place in the story is unusual in that he partakes of few of the play’s good things, its rich clothes and feasts. Yet his identity is formed in relation to the riches that populate the play while he is absent. Other characters become deeply invested in self-fashioning through material goods, and I will argue that Ralph’s sense of self appears all the more lucidly as an alternative to that kind of identity.

Much recent criticism of *Shoemaker*’*s* has centered on the play’s interest in material goods and their production, locating the play in the context of a commercial sea change that saw the importation of large quantities of luxury consumer goods. Natasha Korda, for instance, reads “the sign of the last” as a token of the shoemaker’s trade (the “last” which stretches shoes) and as a play on words—shoemakers as among the last bastions of traditional trade in a London increasingly driven by mass production and importation.^11^ Matthew Kendrick goes further in his reading of the play as an economic fable, claiming that “what we find in the play is a tension between three models of economic and social value: an aristocratic sense of inherent value and social importance, a mercantilist or proto-capitalist vision in which all value is understood in commercial terms, and a counter-discourse which locates the labouring body as the source of value and meaning.”^12^ In this commercial, status-obsessed world, it is fitting that when Ralph is pressed into the army to fight a war in France, his parting gift to the “loving, lovely” Jane (1:224) is a pair of shoes:

Here, take this pair of shoes cut out by Hodge,
Stitched by my fellow Firk, seamed by myself,
Made up and pinked with letters for thy name
Wear them, my dear Jane, for thy husband’s sake,
And every morning, when thou pull’st them on,
Remember me, and pray for my return.
Make much of them, for I have made them so,
That I can know them from a thousand moe.
(1:228–35)

Jane’s shoes are not only a material property; throughout the exchange, they are closely aligned with Ralph’s hand-work and his cooperation with his fellows, Hodges and Firk, as well as his skill at “pinking,” also known as “pricking.” For Jane’s part, the shoes are emblematic of the faithfulness with which she will guard access to her body, despite the jeopardy which attends being left alone in the city, and which leads Firk to suggest she might earn a living as a prostitute (1:137–41). This alignment of goods with bodies and bodily status sets the stage for the action to come, in which the equation between material and personal properties becomes increasingly apparent. Significantly, the shoes are also a collaborative effort between Ralph and his fellows. The capacity of manual skill to bring people together and enable social mobility is repeatedly emphasized in the play. In scene 4 alone, the journeymen and their “Gentle Craft” are described as “fine” eight times, as well as “proper,” “brisk,” “good fellows,” and “true Trojans.” “[T]he play,” in Kendrick’s terms, “subjectivizes labour, giving it a prominent voice and determinant place within the social.”^13^

An emphasis on material goods therefore underlines the place of artisanal economies at the heart of commercial London. More than this, however, the play’s characters—with the notable exception of Ralph—find their identity in the sumptuary. As Kendrick observes, the primacy of handiwork and artisanal skill in *Shoemaker*’*s* makes elision between self and thing particularly easy: “The social world, from the artisanal perspective, is made, not given.”^14^ More generally, the significance of clothes as transformative items in the early modern period has long been recognized by historians of dress. In their *Renaissance Clothing and the Materials of Memory* (2000), for instance, Peter Stallybrass and Ann Rosalind Jones argue persuasively that “To understand the significance of clothes in the Renaissance, we need to undo our own social categories, in which subjects are prior to objects, wearers to what is worn. We need to understand the animatedness of clothes, their ability to “pick up” subjects, to mold and shape them both physically and socially, to constitute subjects through their power as material memories.”^15^ The notion espoused here, that “fashion fashions,” transforming and becoming identical with the body, corresponds closely with readings of material goods as “prostheses,” items that both signal and materialize one’s way of being in the world.^16^ This reading in turn relies on an idea of prostheses, broadly defined, as “supplemental” in a Derridean sense. That is, the items that are added to the body both supply a deficiency and make that deficiency conspicuous, at once transforming the “original” and remaining apart from it. In his *Materialising Gender*, for instance, Will Fisher adopts what he terms a “free-ranging” definition of prosthesis that includes items such as hair, handkerchiefs, and codpieces.^17^ Fisher views these items as “auxiliary organs” mediating the body’s form and function.^18^ He describes the codpiece, for example, as a “constitutive accessory”: “I believe that the codpiece wasn’t simply a sign of masculinity, but that it quite literally helped to mold the body and make the man. Or, to use the language of a writer from the period, the codpiece had the ability to make an individual ‘man in body by attire.’”^19^

In *Shoemaker*’*s*, the idea of sumptuary transformation is played out in spectacular style. With the aid of manual skill “learned in Wittenberg” (3:21), a phony Dutch accent, and a set of new clothes, idle nobleman Lacy is able to disguise himself as Hans the shoemaker. This transformation is not only one of “semblance,” but an alteration of the bodily and mental state, a “change [from] / High birth to baseness” (3:20–21), which Lacy equates with the metamorphoses of the classical gods (3:1–2). Even more noticeable is the play’s obsession with Margery and Eyre’s new clothes—garments that go hand-in-hand with Eyre’s new venture into buying and selling luxury commodities.^20^ Immediately as the couple are attired in fine clothing, they acquire new status in their own eyes and those of onlookers:

Firk. Ha, ha! My master will be as proud as a dog in a doublet, all in beaten damask and velvet.

Eyre. Softly, Firk. For rearing of the nap, and wearing threadbare my garments. How dost thou like me Firk? How do I look, my fine Hodge?

Hodge. Why now you look like yourself, master! I warrant you, there few in the city but will give you the wall, and come upon you with the ‘Right Worshipful’.

Firk. ’Nails, my master looks like a threadbare cloak turned new and dressed. Lord, Lord, to see what good raiment doth!

(7:98–107)

Clothes make the (wo)man in these cases in a profound sense. These garments not only transform the couple in their outer appearances, but are, as Jonathan Gil Harris has commented, envisioned as acting upon the very flesh of their bodies, “not just arous[ing] the characters but ultimately eras[ing] them.”^21^ In characterizing Eyre as looking like a cloak, Firk collapses the distinction between his master and the garment he wears, so that new clothes possess the alluring power to remake one’s essential self. Hodge, meanwhile, responds that “now you look like yourself,” backdating Eyre’s transformation to imply that the new cloak has merely revealed his master’s hidden qualities.

Clothes may also confer power. At the end of the scene, Margery comments, “I do feel honour creep upon me, and which is more, a certain rising in my flesh” (7:134–5). The bawdy subtext of “rising . . . flesh” implies a newfound agency that is so potent it is imagined as phallic. While she may be quick to put on airs, Margery’s assessment of her new “honour” is accurate; by the end of the play, Eyre is Lord Mayor, and she is no longer Dame but “Mistress.” The equation between bodies and garments, between personal and commercial value, takes on a darker side in relation to Ralph’s abandoned wife, Jane. Attempting to woo Jane in the shop where she works, Hammon fails to understand the difference between buying a ruff or a handkerchief and purchasing a wife. When Jane asks “What will you buy?” he replies “That which thou wilt not sell. *Faith, yet, I*’*ll try*” (12:22–23, my emphasis). When she responds negatively to his offer, he redoubles his insistence on the commercial nature of their exchange:

Jane. My hands are not to be sold.

Hammon. To be given, then.

Nay, faith, *I come to buy*.

(12:26–28, my emphasis)

Hammon’s treatment of Jane is not only crassness; he is equally willing to equate his own body with material items, as when he remonstrates, “Look how you wound this cloth, so you wound me” (12:24). Neither are Ralph and Jane themselves entirely immune to this pervasive metaphor, though they ultimately reject the mercantile model of identity. When Ralph discovers Jane’s shoe, he declares of the item that “This is her size, her breadth” (14:46). Later, when they are finally reunited, Jane frames the choice between Ralph and the wealthy Hammon as a choice between fine garments and “humble weeds” (18:57).

The effect of material goods as prosthetic markers of identity is, for Dekker, largely positive. It enables social mobility in a play which is pitched to present mobility as a desirable aim. Nonetheless, such markers are, by their nature, unstable, able to be passed around, lost and gained, and reproduced. Inevitably, the more accessible a social marker is, the more liable it is to be tarnished by association with the “wrong sort” of wearer, hence the period’s persistent anxiety about women’s use of cosmetics and both sexes’ adoption of courtly fashions.^22^ The importance of such goods also inevitably implies that the people they prostheticize are in some sense incomplete or incapable of enacting social transformations without material additions.

Taking “prosthesis” in a broad sense thus exposes the instability of that category in ways that are useful to disability studies scholars as they seek to interrogate the very concept of the “whole” or “deficient” body. Central to such scholarship has been the idea that disability itself is perhaps being made to do ideological and narrative work. David Mitchell and Sharon Snyder’s now-seminal *Narrative Prosthesis* (2000) treats disability as a sort of metaphorical prosthesis, acting on (and in) the “body” of literary texts.^23^ The presence of a disabled character in a narrative, they argue, provides “unusualness.” The anomaly of disability drives forward plots and lends a corporeal grounding through which intangible themes may be discussed: “disability has been used . . . as a crutch on which literary narratives lean for their representational power, disruptive potentiality, and social critique. Yet, at the same time, literature avoids designating disability itself as a source for derisive social myths that need to be interrogated.”^24^ Ralph’s disabled body works as a narrative prosthesis, according to this description, because it facilitates both the subplot of Ralph and Jane’s reunion, and the wider focus on identity with which I argue that the play is concerned. Where, though, do these narrative prostheses leave the disabled user of *actual* prostheses? Talk of material objects as supplemental to the body undermines the notion of *any* body as fixed and complete, since, as Margaret Shildrick argues, “the supplement is necessary in constituting the object as such, and in turn its necessity exposes the undecidable nature of categorical distinctions between self/other, natural/artificial, and so on that are usually taken for granted.”^25^ Writing on the philosophy of prosthetics and embodiment, Shildrick astutely points out that the dependence of humans on items from canes to contact lenses undermines the notion that the body should reliably “end at the skin,” and points instead toward the human self as a Deleuzian *assemblage*. In other words, even normatively bodied persons—in this case, Margery, Eyre, and Lacy—have need of prosthetics, though they use them for purposes of social advancement rather than everyday survival.^26^ It is worth pointing out here that anxieties about supplementarity raised by prosthesis may not necessarily make much difference to the experience of disability. Shildrick acknowledges that despite the indistinction between self and thing implied by our universal reliance on material objects, those with “anomalous corporealities” continue to be associated to a greater extent with material-fleshly “hybridity.”^27^ Likewise, Vivian Sobchack—herself a prosthesis user—cautions against focusing on the theoretical possibilities of the prosthetic to the exclusion of the “literal and material ground of the metaphor.”^28^ In the lived experience of disability, she contends, the prosthesis is unremarkable, and “becomes an object only when a mechanical or social problem pushes it obtrusively into the foreground of the user’s consciousness.”^29^

In social dramas such as those played out in *Shoemaker*’*s*, it is not only the extent but the changeability of one’s prosthetics that is at issue. In her work on the “Cripple” of Thomas Heywood’s *The Fair Maid of the Exchange* (1607), Katherine Schaap Williams demonstrates that the difference between nondisabled and disabled bodies is most visible in one’s ability to assume different identities as the occasion demands. That is, the nondisabled person can pretend to be disabled, but not vice versa. In *Fair Maid*, able-bodied Frank’s ability to assume the “habit” of Cripple enables him to woo Phyllis *and* seamlessly return to the social and economic advantages of the normatively bodied: “Notably, however, in a play that has thematized exchange, the key thing to see is that the exchange is never equal. Cripple cannot participate fully: Frank can impersonate Cripple, but Cripple cannot impersonate Frank. . . . Distinguishing between prop and prostheses at the level of character, the play differentiates between bodies that appear to be whole, self-sufficient, and capable of impersonating other bodies, and those that are not.”^30^ Williams’ thesis is partially applicable to *The Shoemaker*’*s Holiday*. Cripple and Ralph both have bodily differences that become visible in relation to the play’s other characters. However, the degree to which they are thus determined is very different. When Frank impersonates Cripple, he pays attention almost exclusively to Cripple’s disability. Far from seeking to reproduce Cripple’s manner of speech or professional knowledge, he attributes new qualities to his character based solely on his disability, declaring himself “Crooked in shape, and crooked in my thoughts.”^31^ Cripple has never shown any sign of a “crooked” mind, but genuine acquaintance is here overridden by the determining factor of bodily impairment. Ralph too has an identity constructed largely through difference. In this case, however, the result is far hazier. As we have seen, many of *Shoemaker*’*s* characters actively seek to (re)make their identities with the help of material prostheses. By contrast much of Ralph’s character is constructed in the negative; he does not do and acquire the things that his companions place so much emphasis on doing and acquiring. This may be because, as I shall discuss, his disability places him at a socioeconomic disadvantage, or because he expresses little desire for wealth beyond that required to support himself and his wife. Most likely, it is both.

The difference between Ralph’s portrayal and that of other characters is visible from the moment he returns home from the wars in scene 10. Here, the contrast between the overdetermined materiality of Margery, Eyre, and Lacy/Hans’s new identities and the indeterminacy of Ralph’s status is striking. At first the returning soldier is unrecognizable, and when he is recognized, he receives a strange reception:

Hodge. What, fellow Ralph! Mistress, look here—Jane’s husband! Why, how now—lame? Hans, make much of him: he’s a brother of our trade, a good workman, and a tall soldier.

(10:53–55)

This list of Ralph’s roles—husband, brother, worker, soldier—underlines the bonds of fellowship within which the returning journeyman’s identity is constituted, and sounds like an attempt by Hodge to deflect attention from his friend’s altered appearance. Hodge refers to Ralph with the same epithet—tall—as before he left for the wars, though it becomes clear that Ralph’s body is emphatically *not* the same:

Margery. Trust me, I am sorry Ralph, to see thee impotent. Lord, how the wars have made him sunburnt! The left leg is not well; ’twas a fair gift of God the infirmity took not hold a little higher, considering thou camest from France—but let that pass.

(10:61–64)

Margery’s description of Ralph’s injury is the most explicit in the play, but it is still frustratingly vague. We know from Dekker’s text just how to recognize Lacy/Hans in his Dutch attire, and Margery and Eyre in their high heels and cloak, respectively. In Ralph’s case, however, the text is less forthcoming. This passage is usually interpreted as meaning that Ralph has lost all or part of the left leg, which seems a reasonable reading given the frequency with which catastrophic injuries and gangrene were treated by amputation at this time. Lengthy instructions for amputation appear in virtually every book of military surgery of the period, despite the high death rates that attended so radical an operation. The preeminent surgeon of the sixteenth century, Ambroise Paré, recognized that “This remedy is miserable and worthy of compassion . . . but it is the only and last refuge, which one must still prefer to death, which will follow if one seeks other means than section of the mortified part.”^32^

Margery and Eyre’s encounter with Ralph is soon truncated by the news of Eyre’s promotion to Lord Mayor, their halting acknowledgment of Ralph’s altered materiality overtaken by the promise of the new materials that will accompany civic office. Thereafter, the text’s engagement with Ralph’s disability is (like his leg) conspicuous by its absence. When it occurs, it is in the rather discordant form of jokes about his sexual prowess. Perceiving Ralph’s distress at his injured state, Margery puns on the double meaning of “thing”: “He does but as I do, weep for the loss of any good thing” (10:98–99). Firk, meanwhile, repeatedly teases him about his virility:

Thou [Ralph] do for her? Then ’twill be a lame doing: and that she loves not. Ralph, thou mightest have sent her to me: in faith, I would have yerked and firked your Priscilla (13:23–24)

Thou lie with a woman, to build nothing but Cripplegates! (14:66)

Firk’s jests partake in a tradition of curiosity about the sex lives of disabled people with which the audience would have been familiar.^33^ As Farr has noted of the eighteenth century, disabled bodies existed “along a similar social continuum to that of unauthorized genders and sexualities . . . in a liminal space where unmonitored, unmentionable, or merely mockable forms of courtship and sex abound.”^34^ Montaigne’s famous essay “On the lame” demonstrates the extent to which bodily anomaly might be fetishized as particularly apt for the “sport of Venus.”^35^ Nonetheless, these jokes at Ralph’s expense fail to elicit much reaction from the other characters; they are apparently too preoccupied with the business of refashioning themselves, or, more charitably, are embarrassed for the injured man.

If the *text* largely ignores Ralph’s materiality, however, the *play* cannot do the same. Ralph’s missing leg is on stage—or rather, not on stage—whenever he is. So too is the insistent tapping of his crutch on the wooden boards, punctuating the dialogue despite the exclusion of his body from the script. Ralph’s onstage presence thus insistently confirms that difference which his friends are loath to acknowledge. It may also help to explain their lack of acknowledgment. In a society wherein disabled veterans are a significant social and economic problem, Ralph’s body is so visually readable that it shuts down rather than invites comment. In other words, nobody needs to point out that Ralph’s injury renders him vulnerable to poverty and exclusion, since they, he, and the audience are already painfully aware of that fact. With his body so readable and yet unspeakable, Ralph is excluded from the focus on bodily accoutrement and material goods by which the play’s other characters are determined. However, he remains an active character, with whom the audience is invited to sympathize. He must therefore maintain an identity on some other grounds. These grounds, I argue, are the bonds of fraternity and matrimony, the same bonds that are emphasized at Ralph’s impressment, before Lacy’s disguises and the Eyre’s rise to wealth.

In the play’s opening scenes, as I have shown, the bonding properties of shared labor and artisanship are valorized. Moreover, the value of a skilled worker is reiterated in Eyre’s promises to his journeymen and his willingness to accept their demand for a new colleague in Hans. In response to Ralph’s legitimate worries about his economic future, Hodge immediately returns to this language:

Ralph. Where lives my poor heart [Jane]? She’ll be poor indeed Now I want limbs to get whereon to feed.

Hodge. Limbs? Hast thou not hands, man? Thou shalt never see a shoemaker want bread, though he have but three fingers on a hand.

(10:72–76)

Despite certain problems with Hodge’s statement, which I address below, Ralph can and must accept their renewed comradeship:

[. . .] Since I want limbs and lands,
I’ll trust to God, my good friends, and my hands.’
(10:101–2)

Being a man neither of property (lands) nor of parts (limbs), Ralph harks back to the fraternal model which supplements flesh with emotional bonds and skills. Moreover, he does so with remarkable ease. In the face of his real bodily alteration, Ralph’s easy (or hurried) assimilation back into his old role contrasts strikingly with the rigmarole of Lacy’s feigned accent and odd clothes, the Eyres’ putting-on of the trappings of nobility, and Hammon’s efforts to buy a wife like a ruff or a handkerchief. These bodies need constant attention; their appearance, gesture, and status must be diligently fashioned, though their substance is far less radically changed than that of the returning journeyman.

The precedence of relationships to Ralph’s identity comes to the fore during his reunion with Jane. Ralph learns of Jane’s location through the shoe which he made for her, and which is now brought to be copied for her impending marriage to Hammon:

[. . .] this shoe, I durst be sworn,
Once covered the instep of my Jane.
This is her size, her breadth. Thus trod my love.
These true-love knots I pricked. I hold my life,
By this old shoe I shall find out my wife.
(13:44–48)

Ralph gestures here to the equation between things and bodies that has defined other characters’ social positioning: “This is her size, her breadth.” However, he understands the shoe as a separate, lifeless thing, an empty vessel by which he may recover his lost wife. The shoe—tellingly, now “old” despite having been made only weeks or months before—is valuable only as it relates to his marriage and his craft. Encountering his wife as he fits her new shoes, he reports that she has failed to recognize him on account of “my lame leg and my travel beyond sea” (18:11–12). True to form, however, Ralph sees this physical misrecognition as unimportant, since “I know she’s mine” (18:12). He apprehends Jane and Hammon on the way to their wedding, supported by his coworkers and another “five or six shoemakers” seemingly invested in this quarrel by dint of their shared profession with Ralph. In a moving speech, Jane eschews Hammon, explicitly rejecting “his attire,” and returns to her estranged husband (18.55–61).

Ralph, for Jane, is “him whom heaven hath made to be my love” (18.56). As such, they share not only a mutual desire, but a mode of expression which links the pair even when they are far apart. Ralph and Jane are the only two lower-class characters to speak in verse. Ralph slips between prose and verse, using the latter most powerfully when he speaks about his wife in scene 14. Jane, meanwhile, has some of the most eloquent rhymed and unrhymed verse in the play. The couple’s linguistic link echoes their marital one, and aligns Jane with her husband even when she is unaware of his survival (e.g., in scene 12). Verse was also, of course, associated with characters of higher status and gentility, a fact which accords with the story’s roots in a tale of beleaguered nobility. Deloney’s *The Gentle Craft* (1597), from which *Shoemaker*’*s* is adapted, relates the story of brothers Crispin and Crispianus, who, on being exiled from their homeland, both become shoemakers.^36^ Crispin woos a local princess, Ursula, in the course of fitting her shoes, and they are secretly married. Crispianus meanwhile, is pressed into the wars in France, proves his valor in combat, and is knighted. The story is resolved when it is revealed that both brothers are of noble birth. *The Gentle Craft* incorporates numerous motifs of Renaissance romance, including that of a quest away from home and “subject-matter concerning love or chivalry, or both.”^37^ Unsurprisingly, since this story is in turn related to the hagiography of Crispin and Crispinian, the story also dwells on ideals of kinship, bravery, and fidelity.

Dekker adapts rather than retells this story. His version substitutes Lacy for Crispin and retains the pressing of Crispianus (Ralph) into service, but loses both his knighthood and his noble birth. Nonetheless, the romantic motifs of *A Gentle Craft* echo through *Shoemaker*’*s* in the elegant speech of Ralph and Jane. Their relationship, while ostensibly humble, draws on the Homeric notion of a steadfast Penelope, besieged by suitors, awaiting her husband’s return from sea. Jane, in this reading, is both an appropriate wife for a man of valor, and a narrative compensation for the fact that Ralph, unlike Crispianus, does not return home from the wars unharmed and triumphant. The action of *Shoemaker*’*s* thus makes clear Ralph’s devotion to his wife and friends, but the play’s intertextuality, making itself felt through moments of unexpected lyricism, casts Ralph and Jane’s relationship as romantic in both senses of the word.

Ralph’s restoration to the community of shoemakers and to the arms of his loving wife appears to confirm the value of interpersonal relationships over and above material goods. In what Kastan describes as an example of “wish-fulfilling logic,” the pair even get to keep the money and clothes offered to Jane by Hammon.^38^ Still, there are some nagging aspects of Ralph’s happy ending that suggest this solution may not be as neat as it appears. Ralph’s fellow shoemakers have neglected his explicit charge to protect Jane by allowing her to be bullied out of the shop by Margery. Even in the face of his obvious distress, they are unwilling to admit their fault. Moreover, as Lindsey Row-Heyveld has pointed out, Ralph’s reintegration has its limits. Margery casts doubt on Ralph’s sexual and economic usefulness when she describes him as impotent. Moreover, when Hodge enters the shop decked in a gold chain in scene 10, he does not acknowledge Ralph, much less offer him any share in his newfound wealth. Row-Heyveld concludes: “Ralph may be offered charity, but, as with the loss of Jane, it is a charity with distinct limits; Ralph may be welcomed back into the cadre of shoemakers, but it is clear that his disability shifts their interpretation of his commercial and sexual labor, and, therefore, segregates him from his ‘brothers.’ Even within the play’s fantasy of masculine unity, divisions between men—especially those created by disability—never fully disappear.”^39^ Why does Dekker not follow his “wish-fulfilling logic” to secure an unequivocally warm reception for Ralph? Why also allow Ralph to be insulted by another man offering money for his wife? I contend that the ambiguity which remains around Ralph’s reintegration works as tacit acknowledgment of the junction between Dekker’s use of disability on stage as a tool for thinking about identity, and the fact of disability as it was lived by members of the audience. The actor who played Ralph feigned lameness for dramatic purposes, but he played to people for whom impairment may have been anything but imaginary. Sobchack describes this junction eloquently: “there are both an *oppositional tension* and a *dynamic connection* between the prosthetic as a tropological figure and my prosthetic as a material but also a phenomenologically lived artifact.”^40^ In the early modern period, this tension and connection were present, and pressing. War plays were popular, and depictions of martial peril included the dangers of war wounding alongside the potential for honor.^41^ Christopher Morrow describes lame soldiers as having been “a staple on the Renaissance stage,”^42^ while Cahill identifies at least eight plays published or performed between the late 1590s and 1602 featuring a lame soldier.^43^ Perhaps to a greater extent than before or since, these plays were viewed by people who had actually seen, and been disabled by, military service, men who looked more like Ralph than his companions.^44^ In his *The Seat of Mars*, Charles Carlton estimates that under Elizabeth, around 55 percent of all males between the ages of 18 and 39 saw some kind of military service.^45^ Like Ralph, a significant minority of soldiers were forced into fighting; the numbers of those impressed by the military rose sharply from 4,835 in 1597 to 7,300 in only the first half of 1599.^46^ Messy, prolonged, and often poorly organized, these conflicts produced large numbers of wounded as well as fatalities. War had always created casualties, but the increased use of firearms and artillery from the mid-sixteenth century onward inevitably resulted in more catastrophic and complex injuries for which the only remedy was radical surgery.^47^ The situation was so grave that only seven years before the first performances of *The Shoemaker*’*s Holiday*, the “Act for the Necessary Relief of Soldiers and Mariners” was passed in order to alleviate the plight of those who returned from wars unable to work and were forced to beg in order to survive.^48^ Moreover, despite the formalization of relief for maimed soldiers provided by the Act, it is clear that wounded veterans faced an uncertain fate, struggling to avoid being lumped in with vagrants or mistaken for malingerers and to emphasize their deserving status.^49^

This brings one to a fundamental irony of prosthetics and identity in *Shoemaker*’*s*. Ralph, as I have shown, is the character whose body most “speaks for itself” and the one who makes least use of sumptuary prostheses. Yet Ralph’s is also the body on stage which is least “like itself,” because his disability is “put on” by an able-bodied actor.^50^ How exactly this was achieved is uncertain. Farah Karim-Cooper has recently shown how hand amputation might be represented on stage using a wax or wood prosthetic that was lopped off, leaving the actor’s real hand inside the sleeve.^51^ A similar effect could be used for leg amputation, with the actor’s leg bent double inside his breeches or slops.^52^ As Vin Nardizzi has speculated, Ralph’s lameness might thus be represented with his leg absent and the actor balancing with a crutch, with a wooden peg leg, or with an “injured” prosthetic leg.^53, 54^ The fact that Dekker left the exact nature of Ralph’s injury unstated was perhaps a deliberate move designed to allow theatrical companies to stage *Shoemaker*’*s* according to the props and actors they had available.

What is clear, however, is that the actor’s putting-on of disability in general, and in *Shoemaker*’*s* in particular, troubles the always-tremulous distinction between props and prosthetics, between objects that disguise the body and objects that transform it, and between playing a role on stage and in daily life. Describing this phenomenon as “disability drag,” Lauren Coker-Durso argues that seeing the same actor in disabled and nondisabled roles created the impression of disability as something that might be removed at will: “The performance of disability and fraudulent disability in non-dramatic settings works in a dialogue with the onstage interpretations in historical and cultural contexts. An openly acknowledged performance often portrays disability as a choice, as something autonomously constructed. In other words, deliberately staged enactments of disability in dramatic texts foster a cultural push towards denying or questioning disability as a material corporeality in various contexts. In many ways, disability is constructed as *only* performance.”^55^ Early modern performances of disability might thus be said to create some of the same problems as modern “super-crip” narratives, which present disabled people as overcoming their impairment to achieve heroic feats. While they have the potential to change public attitudes toward disability, such stories all too often elide the social and economic barriers affecting many people with disabilities, and may imply that all disability can be overcome with sufficient effort.^56^

A somewhat puzzling moment in Dekker’s play confronts directly the uncomfortable juncture between disability drag and the real lives of disabled people. Shortly after their reunion, Ralph and Jane—he, lame, and she wearing a veil—are accosted by Oatley and Lincoln, who assume first that his lameness is feigned, and second that her veil constitutes an attempt to counterfeit blindness (18.114–40). They are soon proved wrong, but the older men’s assumption is founded in a widespread suspicion of disability that was fueled by literary and theatrical representations of impairment. Audrey Eccles, Linda Woodbridge, and Row-Heyveld, among others, have demonstrated how suspicion of disabled people in general, and the disabled poor in particular, reached fever pitch in the sixteenth and seventeenth centuries, driven by legislative and economic changes that formalized the obligation of communities toward their “impotent” members and clamped down on the itinerant poor.^57^ Endless medical texts and cony-catching pamphlets detail the nefarious means used by vagrants to fake sores, amputations, and blindness—means which, ironically, may have brought about real illness and injury.^58^ The ruses described in such texts were inventive and frequently theatrical in nature. Paré, for instance, recalled having seen (and exposed) a number of remarkable instances of illness-shamming, including fake cancerous ulcers made of sponge, animal blood, and frog skins, a woman feigning a prolapsed bowel using cow intestines, and a man who stole a decaying arm from a corpse to pretend it was his own diseased limb.^59^ Accordingly, a large proportion of disability represented on stage was *faked* disability. Row-Heyveld identifies over 30 characters in early modern plays who pretend to be disabled, a trend which she argues was exacerbated by a post-Reformation shift away from the semi-reciprocal, “alms-for-prayers” mode of charity.^60^ Furthermore, it was believed that both disabled and able-bodied vagrants commonly masqueraded as discharged or wounded soldiers in order to access the public goodwill and official lenity that was (sometimes) afforded to that group. In the 1616 edition of Dekker’s own *O per Se O*, he detailed at length how unscrupulous beggars faked soldiers’ passports and created sores from lime and soap.^61^

Disability and theatricality thus constantly informed one another, as actors feigning disability played to audiences including disabled people who might or might not be “acting” their impairment, and able-bodied people for whom the spectacles of real and faked injury were worryingly indistinct. Depictions of fakery made life harder for genuinely disabled people, not only because it rendered them objects of suspicion, but because the intense focus on verifying or falsifying impairment allowed able-bodied people to avoid thinking about more troublesome matters. While wondering about the mechanics of feigning a missing leg, onlookers were not addressing the significant hardships faced by the genuinely impaired, or recognizing their own vulnerability to disabling accidents and illness. Dekker’s hesitation to complete the wish fulfilment of *Shoemaker*’*s* therefore recognizes the contradiction between presenting one sympathetic disabled character and reaffirming the belief that disability might be put on. By muddying the waters around Ralph’s happy ending, by recognizing the fragility of his reintegration, Dekker recognizes that the disability he employs on stage as a storytelling tool and that which actually affects members of the audience have an important but difficult relation to one another.

## Conclusion

*The Shoemaker*’*s Holiday* epitomizes the puzzle of disability on the Renaissance stage. On one hand, this is a play about being oneself. Dekker makes clear that Ralph, whose body is “lacking,” nonetheless resists the prostheticized identity which other characters embrace. Margery, Eyre, and Lacy fashion themselves through material goods, which both express and transform their social and economic status. By contrast, Ralph uses a traditional prosthesis, with an inescapable auditory and visual presence on stage. However, the things that make Ralph *Ralph* are those which persist despite his prosthetic alteration—fellowship, work, and marital harmony. On the other hand, we might argue that “Ralph” is not really Ralph at all. In fact he is, physically, further from being Ralph than the other actors are from being Eyre or Firk, and Ralph’s onstage body is far more analogous to the offstage bodies of certain members of the audience. This fact brings home the constant tension between lying, playing, and truly presenting oneself that troubles both the early modern stage in general and the issue of representing disability in particular.

Both these considerations can, I think, coexist, and the play is more interesting for acknowledging this puzzle. The fact that Ralph’s disability is acted does not render the play’s emphasis on his alternative ontology obsolete, any more than the fact of boy actors renders presentations of the lives of women untenable. Audiences suspend disbelief in the real physical attributes of characters and actors just as they do in cases of magic, unlikely circumstance, or indeed incredible social climbing. Nonetheless, Dekker’s optimistic presentation of a disabled veteran reconstructing his identity in interpersonal terms takes place in an environment in which the distinction between disguised and transformed bodies is decidedly vague, and in which the theatricality with which disability is imbued does real harm to those suffering from illness and injury. The result is a play that heralds with optimism the possibility of creating genuine relationships based on empathy and personality, but acknowledges the difficulties encountered by those living with an anomalous body.

## Footnotes

1 All references to *The Shoemaker*’*s Holiday* are given in-text as “scene:line” (the play is not divided into acts), and use Thomas Dekker, *The Shoemaker*’*s Holiday*, ed. Jonathan Gil Harris, 3rd ed. (London: Bloomsbury, 2008).

2 *Henslowe*’*s Diary* (London: A. H. Bullen, 1904), 37, http://archive.org/details/cu319240 26121305.

3 In Gil Harris’s edition of the play, he modernizes the original “Rafe” to “Ralph,” and I follow that spelling here.

4 “Introduction,” in Dekker, *The Shoemaker*’*s Holiday*, ix–x.

5 Michael Manheim, “The Construction of *The Shoemakers*’ *Holiday*,” *Studies in English Literature, 1500*–*1900* 10, no. 2 (April 1970): 315–23.

6 Peter Mortenson, “The Economics of Joy in The Shoemakers’ Holiday,” *Studies in English Literature, 1500*–*1900* 16, no. 2 (1976): 241, doi:10.2307/449765.

7 Christopher L. Morrow, “Corporate Nationalism in Thomas Dekker’s The Shoemaker’s Holiday,” *SEL Studies in English Literature 1500*–*1900* 54, no. 2 (2014): 423–54, doi:10.1353/sel.2014.0014.

8 Matthew Kendrick, “‘A Shoemaker Sell Flesh and Blood—O Indignity!’: The Labouring Body and Community in The Shoemaker’s Holiday,” English Studies 92, no. 3 (May 2011): 259–73, doi:10.1080/0013838X.2011.564779; Natasha Korda, “‘The Sign of the Last’: Gender, Material Culture, and Artisanal Nostalgia in *The Shoemaker*’*s Holiday*,” *Journal of Medieval and Early Modern Studies* 43, no. 3 (September 2013): 573–97, doi:10.1215/10829636-2338608.

9 Patricia Cahill’s doctoral thesis, “‘Tales of Iron Wars’: Martial Bodies and Manly Economies in Elizabethan Culture” (2000), is a notable exception. The thesis was published in 2008 as *Unto the Breach: Martial Formations, Historical Trauma, and the Early Modern Stage* (Oxford: Oxford University Press, 2008), with a curtailed discussion of *The Shoemaker*’*s Holiday*.

10 David Scott Kastan, “Workshop and/as Playhouse: Comedy and Commerce in ‘The Shoemaker’s Holiday,’” *Studies in Philology* 84, no. 3 (1987): 324–37.

11 Korda, “The Sign of the Last.”

12 Kendrick, “A Shoemaker Sell Flesh and Blood—O Indignity!,” 260.

13 Ibid., 273.

14 Ibid., 272.

15 Ann Rosalind Jones and Peter Stallybrass, *Renaissance Clothing and the Materials of Memory* (Cambridge: Cambridge University Press, 2000), 2. See also Edith Snook, *Women,* Beauty and Power in Early Modern England (London: Palgrave Macmillan UK, 2011), in which Snook argues that clothes “not only signal a woman’s inwardness but also actively engender it” (68).

16 Jones and Stallybrass, *Renaissance Clothing*, 3.

17 Will Fisher, *Materializing Gender in Early Modern English Literature and Culture* (Cambridge: Cambridge University Press, 2006), 29.

18 Ibid., 27.

19 Will Fisher, “‘Had It a Codpiece, ’Twere a Man Indeed’: The Codpiece as Constitutive Accessory in Early Modern English Culture,” in *Ornamentalism: The Art of Renaissance Accessories*, ed. Bella Mirabella (Ann Arbor: University of Michigan Press, 2011), 103.

20 In scene 9, it is revealed that Eyre profits substantially from his investment: “Eyre’s gains in one commodity / Rise at the least to full three thousand pound, / Besides like gain in other merchandise.” Dekker, *The Shoemaker*’*s Holiday*, 9:68–70.

21 Ibid., xv.

22 See Mark Breitenberg, Anxious Masculinity in Early Modern England (Cambridge: Cambridge University Press, 1996), 73–74; John Gauden, *A Discourse of Auxiliary Beauty. Or Artificiall Hansomnesse. In Point of Conscience between Two Ladies* (London: printed for R. Royston, 1656); Snook, *Women, Beauty and Power*, 21–26.

23 David T. Mitchell and Sharon L. Snyder, *Narrative Prosthesis: Disability and the Dependencies of Discourse* (Ann Arbor: University of Michigan Press, 2000).

24 David T. Mitchell, “Narrative Prosthesis and the Materiality of Metaphor,” in *Disability Studies: Enabling the Humanities*, ed. Sharon L. Snyder, Brenda Jo Brueggemann, and Rosemarie Garland-Thomson (New York: Modern Language Association of America, 2002), 17.

25 Margaret Shildrick, “‘Why Should Our Bodies End at the Skin?’: Embodiment, Boundaries, and Somatechnics,” Hypatia 30, no. 1 (February 2015): 17, doi:10.1111/hypa.12114.

26 On this subject, see also Cassandra S. Crawford, Phantom Limb: Amputation, Embodiment, and Prosthetic Technology (New York: NYU Press, 2014); Patrick Schmidt, “No Need for Assimilation? Narratives about Disabled Persons and Their Social Integration in Eighteenth-Century Periodicals,” in *Imperfect Historian: Disability Histories in Europe*, ed. Sebastian Barsch, Anne Klein, and Pieter Verstraete (Frankfurt: Peter Lang AG, 2013), 41–59; David M. Turner and Kevin Stagg, *Social Histories of Disability and Deformity: Bodies, Images and Experiences* (Abingdon: Taylor & Francis, 2006).

27 Shildrick, “Why Should Our Bodies End at the Skin?,” 16.

28 Vivian Sobchack, “A Leg to Stand On: Prosthetics, Metaphor, and Materiality,” in *The Prosthetic Impulse: From a Posthuman Present to a Biocultural Future*, ed. Marquard Smith and Joanne Morra (Cambridge, MA: MIT Press, 2006), 20. See also Vivian Sobchack, “Living a ‘Phantom Limb’: On the Phenomenology of Bodily Integrity,” Body and Society 16, no. 3 (September 2010): 51–67, doi:10.1177/1357034X10373407.

29 Sobchack, “A Leg to Stand On,” 22.

30 Katherine Schaap Williams, “‘More Legs than Nature Gave Thee’: Performing the Cripple in The Fair Maid of the Exchange,” ELH 82, no. 2 (2015): 507.

31 Thomas Heywood, *The Fayre Mayde of the Exchange: With the Pleasaunt Humours of the Cripple of Fanchurch. Very Delectable, and Full of Mirth*, 13317 (STC 2nd ed.) (London: Printed by Valentine Simmes for Henry Rockit, and are to be solde at the shop in the Poultrey vnder the Dyall, 1607), fol. H3v.

32 Ambroise Paré, *Ten Books of Surgery with the Magazine of the Instruments Necessary for It*, fac-simile reprint (Athens: University of Georgia Press, 2010), 134.

33 See David M. Turner, “Introduction: Approaching Anomalous Bodies,” in Turner and Stagg, *Social Histories of Disability and Deformity*, 4. For an example of curiosity about the sexual activities of “cripples,” see Anon., *Deeds against Nature, and Monsters by Kinde Tryed at the Goale Deliuerie of Newgate, at the Sessions in the Old Bayly, the 18. and 19. of Iuly Last, 1614. the One of a London Cripple Named Iohn Arthur, That to Hide His Shame and Lust, Strangled His Betrothed Wife*. . . . *With Two Sorrowfull Ditties of These Two Aforesaid Persons, Made by Themselues in Newgate, the Night before Their Execution*, Early English Books, 1475–1640 / 908:06 (London: Printed [by G. Eld] for Edward Wright, 1614).

34 Jason S. Farr, “Queer Deformities: Disability and Sexuality in Eighteenth-Century Women’s Fiction—Haywood, Scott, Burney” (ProQuest Dissertations Publishing, 2013), 7.

35 Michel Montaigne, “On the Lame,” in *The Complete Essays*, trans. M. A. Screech (London: Penguin UK, 1993), 1160–73.

36 Thomas Deloney, *Deloney*’*s Gentle Craft*, ed. Wilfrid J. Halliday (Oxford: Oxford University Press, 2012).

37 Helen Cooper, The English Romance in Time: Transforming Motifs from Geoffrey of Monmouth to the Death of Shakespeare (Oxford: Oxford University Press, 2004), 11.

38 Kastan, “Workshop and/as Playhouse,” 331.

39 Lindsey Dawn Row-Heyveld, “Dissembling Disability: Performances of the Non-standard Body in Early Modern England” (thesis, University of Iowa, 2011), 150, http://ir.uiowa.edu/etd/4906/.

40 Sobchack, “A Leg to Stand On,” 18.

41 Adam N. McKeown, *English Mercuries: Soldier Poets in the Age of Shakespeare* (Nashville: Vanderbilt University Press, 2009).

42 Morrow, “Corporate Nationalism,” 439.

43 Cahill, “Tales of Iron Wars,” 2000), 199.

44 Nick De Somogyi, *Shakespeare*’*s Theatre of War* (Farhnam: Ashgate, 1998), 4–5.

45 Charles Carlton, *This Seat of Mars: War and the British Isles, 1485*–*1746* (New Haven, CT: Yale University Press, 2011), 55.

46 Morrow, “Corporate Nationalism,” 427.

47 James Raymond, *Henry VIII*’*s Military Revolution: The Armies of Sixteenth-Century Britain and Europe*, International Library of Historical Studies 43 (London: Tauris Academic Studies, 2007), 42.

48 Audrey Eccles, *Vagrancy in Law and Practice under the Old Poor Law* (Farhnam: Ashgate, 2012), 2–3; Sarah Ingham, *The Military Covenant: Its Impact on Civil*–*Military Relations in Britain* (London: Routledge, 2016), 31–32.

49 Anne Borsay, “Returning Patients to the Community: Disability, Medicine and Economic Rationality before the Industrial Revolution,” *Disability and Society* 13, no. 5 (November 1998): 645–63, doi:10.1080/09687599826443; Anne Borsay and Peter Shapely, eds., *Medicine, Charity and Mutual Aid: The Consumption of Health and Welfare in Britain, c. 1550*–*1950*, Historical Urban Studies (Farnham: Ashgate, 2007).

50 With the possible exception, of course, of Margery and Jane’s “female” bodies, which are represented by boy actors. “Putting on” gender and disability are two practices with much in common, as Coker-Durso’s term “disability drag” has recognized. Lauren G. Coker-Durso, “Metatheatricality and Disability Drag: Performing Bodily Difference on the Early Modern English Stage” (PhD dissertation, Saint Louis University, 2014).

51 Farah Karim-Cooper, *The Hand on the Shakespearean Stage: Gesture, Touch and the Spectacle of Dismemberment* (London: Bloomsbury, 2016), 219–21.

52 This must have been the technique used in *Doctor Faustus* (act 4, scene 6), in which Faustus has his leg pulled off while he sleeps. Christopher Marlowe, *The Tragical History of Doctor Faustus*, in *Christopher Marlowe: The Complete Plays*, ed. J. B. Steane (Aylesbury: Penguin, 1977), 259–340.

53 Vin Nardizzi, “The Wooden Matter of Human Bodies: Prosthesis and Stump in *A Larum for London*,” in *The Indistinct Human in Renaissance Literature*, ed. Jean E. Feerick and Vin Nardizzi, Early Modern Cultural Studies (New York: Palgrave Macmillan US, 2012), 129–30.

54 Modern productions of *The Shoemaker*’*s Holiday* have depicted Ralph in various states of disability and deformity. The Royal Shakespeare Company’s December 2014–March 2015 production shows the returning soldier limping heavily, with a crutch, and with burns to one side of his face. In Philip Dexter’s 1981 National Theatre production, Ralph’s return is similarly exploited for dramatic effect: he is seen at a half-open stable door, the lower half of which is then thrown back to reveal that he has only one leg. Mark Amory, “Such Dexterity,” *Spectator Archive*, June 27, 1981; Dekker, *The Shoemaker*’*s Holiday*, xxvii.

55 Coker-Durso, “Metatheatricality and Disability Drag,” 10.

56 On this subject, see Sami Schalk, “Reevaluating the Supercrip,” Journal of Literary and Cultural Disability Studies 10, no. 1 (March 2016): 71–86, doi:10.3828/jlcds.2016.5.

57 Row-Heyveld, “Dissembling Disability”; Eccles, *Vagrancy in Law and Practice*; Linda Wood-bridge, *Vagrancy, Homelessness, and English Renaissance Literature* (Urbana: University of Illinois Press, 2001). See also David Turner, *Disability in Eighteenth-Century England: Imagining Physical Impairment* (New York: Routledge, 2012).

58 Aside from the risk of disease associated with the materials used to fake illness, such incidents garnered harsh punishment. Paré reported that one man found to have been faking leprosy was beaten on three consecutive Sundays and subsequently died. Wallace B. Hamby, *The Case Reports and Autopsy Records of Ambroise Paré* (Springfield, IL: Charles C. Thomas, 1960), 118–19.

59 Ibid., 116–19. See also David Turner, “Disability Humor and the Meanings of Impairment in Early Modern England,” in *Recovering Disability in Early Modern England*, ed. Allison P. Hobgood and David Houston Wood (Columbus: Ohio State University Press, 2013), 57–72.

60 Row-Heyveld, “Dissembling Disability,” 1.

61 *O per Se O. Or A New Cryer of Lanthorne and Candle-Light Being an Addition, or Lengthening, of the Bell-Mans Second Night-Walke. In Which, Are Discouered Those Villanies, Which the Bell-Man (Because Hee Went I*’*th Darke) Could Not See: Now Laid Open to the World. Together with the Shooting through the Arme, Vsed by Counterfeit Souldiers: The Making of the Great Soare, (Commonly Called the Great Cleyme:) The Mad-Mens Markes: Their Phrase of Begging: The Articles and Oathes Giuen to the Fraternitie of Roagues, Vagabonds, and Sturdy Beggers at Their Meetings. And Last of All, a New Canting-Song*, Early English Books, 1475–1640 / 880:20 (London: printed [by Thomas Snodham] for John Busbie, and are to be sold at his shop in S. Dunstans Church-yard in Fleet-street, 1612).

